# The treatment and research for posttraumatic stress disorder with Chinese medicine

**DOI:** 10.3402/ejpt.v5.26524

**Published:** 2014-12-09

**Authors:** Yong-Hua Zhang

**Affiliations:** The Seventh Hospital of Hangzhou, Hangzhou, People's Republic of China

**Keywords:** TCM, PTSD, trauma, depression, research

## Abstract

There is no disease called posttraumatic stress disorder (PTSD) in traditional Chinese medicine (TCM). However, Huangdi's Canon of Medicine written in about 200 BC, one of the most famous TCM classics, recorded diseases with similar etiology, pathogenesis, and clinical symptoms. Moreover, contemporary TCM also attaches great importance to diseases caused by trauma. Especially after 2008, there is a mini-rush of study on PTSD as a result of Sichuan earthquake. Referring to ancient and modern literature, we summarize the TCM treatment of PTSD and wish to contribute to the further study on TCM remedy for PTSD.

